# Targeting Lipid Metabolism for the Treatment of Age-Related Macular Degeneration: Insights from Preclinical Mouse Models

**DOI:** 10.1089/jop.2021.0067

**Published:** 2022-01-28

**Authors:** Michael Landowski, Catherine Bowes Rickman

**Affiliations:** ^1^ Department of Medical Genetics, University of Wisconsin-Madison, Madison, Wisconsin, USA.; ^2^ McPherson Eye Research Institute, University of Wisconsin-Madison, Madison, Wisconsin, USA.; ^3^ Department of Ophthalmology and Visual Sciences, University of Wisconsin-Madison, Madison, Wisconsin, USA.; ^4^ Department of Ophthalmology and Duke University Medical Center, Durham, North Carolina, USA.; ^5^ Department of Cell Biology, Duke University Medical Center, Durham, North Carolina, USA.

**Keywords:** mouse models, lipid metabolism, complement, therapeutics, age-related macular degeneration, retinal pigmented epithelium

## Abstract

Age-related macular degeneration (AMD) is a major leading cause of irreversible visual impairment in the world with limited therapeutic interventions. Histological, biochemical, genetic, and epidemiological studies strongly implicate dysregulated lipid metabolism in the retinal pigmented epithelium (RPE) in AMD pathobiology. However, effective therapies targeting lipid metabolism still need to be identified and developed for this blinding disease. To test lipid metabolism-targeting therapies, preclinical AMD mouse models are needed to establish therapeutic efficacy and the role of lipid metabolism in the development of AMD-like pathology. In this review, we provide a comprehensive overview of current AMD mouse models available to researchers that could be used to provide preclinical evidence supporting therapies targeting lipid metabolism for AMD. Based on previous studies of AMD mouse models, we discuss strategies to modulate lipid metabolism as well as examples of studies evaluating lipid-targeting therapeutics to restore lipid processing in the RPE. The use of AMD mouse models may lead to worthy lipid-targeting candidate therapies for clinical trials to prevent the blindness caused by AMD.

## Introduction

Age-related macular degeneration (AMD) affects about 30% of Americans over age 70 and is the most common cause of irreversible blindness among elderly people in industrialized countries.^1–4^ AMD is characterized by the progressive deterioration of the macula, an anatomical region of the retina that contains the highest density of cone photoreceptors and is responsible for visual acuity.^5^ Currently, there are limited interventions that slow or prevent the progression of AMD to its blinding late stages, thus there is an urgency to identify therapies that prevent or delay its progression. AMD is a complex, progressive, retinal degenerative disease influenced by both environmental and genetic factors and is dependent upon advanced age.^6^ It is imperative to devise therapeutic strategies for AMD and curb its burden on societies since the prevalence of AMD is expected to substantially increase in the next decades.^1^

An ideal strategy to treat AMD would involve targeting the early/intermediate “dry” stages before irreparable visual loss occurs in patients. The early stages of AMD are fairly benign and consist of impaired dark adaptation that corresponds to rod photoreceptor dysfunction,^7^ focal accumulation of intermediate-sized (63 to 125 μm diameter) lipid, lipoprotein, and protein-containing deposits known as drusen within Bruch's membrane (BrM),^8^ and choriocapillaris dropout.^9^ Intermediate AMD comprises retinal pigmented epithelium (RPE) pigmentary changes^10^ and larger drusen (>125 μm diameter).^8^ As AMD progresses to its late stages, it is classified as either exudative or nonexudative AMD. The presence of choroidal neovascularization (CNV) characterizes the exudative or “wet” form of late AMD where the formation of immature blood vessels from the choroid advances into the RPE and subretinal space causing fibrosis and scarring and irreparable vision loss.^11^ The nonexudative or “dry” form of late AMD is distinguished by geographic atrophy where there is central loss of RPE and photoreceptors without the presence of vascular leakage.^6^ Most of the AMD-afflicted population has early/intermediate or late “dry” AMD, while 10% of patients present with “wet” AMD.^6^

Current therapies for AMD target pathologies associated with the wet form of AMD, whereas no therapies exist for early/intermediate dry AMD or geographic atrophy. The current gold standard for the treatment of wet AMD is intravitreal injection of antivascular endothelial growth factor (VEGF) antibodies, such as bevacizumab (Avastin) and ranibizumab (Lucentis) or the soluble decoy receptor targeting VEGF-A, aflibercept (Eylea).^12,13^ However, not all wet AMD patients respond well to anti-VEGF therapies.^14^ It is also possible for patients with exudative AMD to progress to late stages of nonexudative AMD even after anti-VEGF treatment.^15^ Currently, the only intervention available for the treatment of dry AMD is Age-Related Eye Disease Supplement (AREDS), an oral supplement containing vitamin C, vitamin E, lutein/zeaxanthin, and zinc. AREDS enhances protection against oxidative stresses in the eye and was shown in the AREDS trials to reduce the risk of advanced AMD by about 25% over a 5-year period in participants with intermediate AMD, although there was no effect in participants with early or no AMD.^16,17^

Development of novel therapies targeting dry AMD should be facilitated by identifying and examining the pathobiological processes implicated in AMD. Inflammation, complement dysregulation, oxidative stress, extracellular matrix (ECM) remodeling, dysregulated lipid metabolism, and angiogenesis have been implicated as key pathobiological mechanisms underlying AMD development and progression.^11^ Polymorphisms in genes that regulate complement activation and lipid metabolism are among the biggest genetic risk factors for AMD.^18^ Strong evidence based on biochemical, genetic, and cell biological studies implicates the alternative pathway of complement in the development of AMD.^18,19^ Growing evidence from studies using preclinical AMD mouse models further supports an important role of dysregulated lipid metabolism in AMD-like pathology development *in vivo* and provides a potential link between complement factors and lipoprotein accumulation and clearance.^20–23^ In this review, we present an overview of mouse models of AMD, present new strategies for modulating lipid metabolism based on findings from AMD mouse model studies, and summarize studies that evaluated treatments focused on lipid metabolism in AMD mouse models. For more comprehensive reviews of lipid metabolism in AMD, we refer readers to these excellent reviews.^24–27^ In addition, we direct readers to this excellent review that highlights higher order animal models of AMD as these models will not be discussed in this review.^28^

## Associations of Dysregulated Lipid Metabolism with AMD

**FIG. 1.**
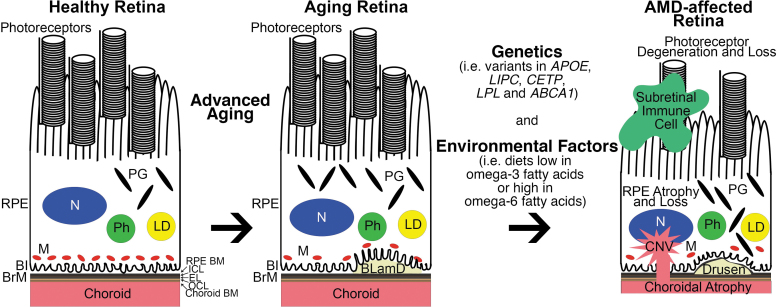
Association of dysregulated lipid metabolism with AMD development and progression. *Left:* Normal, healthy retina. *Middle:* Retina with signs of benign aging such as loss of mitochondria, RPE pigmentary changes, and BLamD formation between the RPE and BrM. *Right:* Through a complex interplay between genetic variants and environmental factors, AMD can develop and progress in the aging retina. Classic pathological hallmarks of AMD include recruitment of subretinal immune cells, photoreceptor degeneration, RPE atrophy and loss, drusen (which start out as basal linear deposits), and form within the ICL of BrM, choroidal atrophy, and CNV. Genetic and epidemiological studies of AMD patients revealed dysregulated lipid transport and metabolism as a key pathobiological mechanism behind AMD. Coding variants in *APOE*, *LIPC*, *CETP*, *LPL*, and *ABCA1* are associated with AMD risk, but it is still unknown why particular variants are linked with disease risk. In addition, diets enriched in omega-6 fatty acids while those deficient in omega-3 fatty acids also increase an individual's risk for AMD. It is widely accepted that omega-6 fatty acids promote, and omega-3 fatty acids dampen retinal inflammation. *ABCA1*, ATP-binding cassette subfamily A member 1; AMD, age-related macular degeneration; *APOE*, Apolipoprotein E; BI, basal infoldings; BLamD, basal laminar deposit; BrM, Bruch's membrane; *CETP*, cholesteryl ester transfer protein; Choroid BM, choroid basement membrane; CNV, choroidal neovascularization; EL, elastic layer; ICL, inner collagenous layer; LD, lipid droplet; *LIPC*, hepatic lipase; *LPL*, lipoprotein lipase; M, mitochondria; N, nucleus; OCL, outer collagenous layer; PG, pigment granule; Ph, phagosome; RPE BM, RPE basement membrane; RPE, retinal pigmented epithelium.

Risk factors for developing AMD include advanced aging, genetic variants, and environmental stressors. Advanced aging is the strongest associated risk factor for AMD (Fig. 1).^29–31^ An individual's risk for developing AMD at age 55 is 0.7%, but this exponentially increases to 22.5% by the age of 80.^32^ Aging induces a number of changes in the retina, including loss of mitochondria,^33^ RPE pigmentary changes,^10^ and accumulation of lipid within BrM, a pentalaminar ECM that separates the RPE from its adjacent choroidal blood supply and acts as a basement membrane for the RPE and choroid (Fig. 1).^34^ The age-dependent accumulation of lipid in BrM is thought to contribute to the development of drusen.^34^ While the presence of a few small “hard” drusen or basal laminar deposits (BLamD) is a normal, nonvision-impairing part of aging, the deposition of large diffuse drusen, or basal linear deposits, in the macula is vision impairing and indicative of intermediate AMD.^34^

Lipid accumulation and cholesterol have long been implicated in AMD disease development.^35,36^ Historically, a role for lipids in AMD has been established through pathohistological examinations of drusen in human donor eyes.^36^ Immunofluorescence, mass spectrometry, and transcript studies of human donor eyes identified apolipoprotein E (ApoE) as a major drusen constituent.^37–41^ ApoE is a protein component of most lipoproteins, lipid-, and protein-containing complexes responsible for packaging cholesterol and fats from the circulation for transport to tissues and removing cholesterol and lipids from tissues through reverse cholesterol transport. The function of ApoE in lipoproteins is to facilitate their cellular uptake as well as bind to extracellular matrices for efficient delivery and removal of lipids from cells.^42^ In addition to ApoE, a number of lipid species such as esterified cholesterol,^40,43,44^ unesterified cholesterol,^40,44^ phosphatidylcholine,^40^ triglycerides,^40^ sphingomyelin,^40^ and fatty acids,^40^ as well as other apolipoproteins^40,41^ have been identified as drusen components. Many of these drusen components are oxidized,^45^ and these oxidized lipids and proteins are believed to contribute to a proinflammatory environment that accelerates AMD development.^46^

The origin of ApoE-containing lipoproteins in drusen is from systemic sources through the circulation and from local synthesis by the RPE, although the relative contribution of each is not known. Circulating lipoproteins must enter and pass through BrM, which is thought to allow passive diffusion of high-density lipoproteins (HDLs) and low-density lipoproteins (LDLs) based on hydraulic conductivity and LDL diffusion studies using bovine BrM explants.^47^ Intriguingly, increased plasma HDLs^48–52^ and LDLs^53^ have been associated with increased risk for AMD and may directly impact drusen accumulation and growth. Evidence that locally derived ApoE-containing lipoproteins contribute to drusen biogenesis comes from studies showing that human RPE cells are capable of synthesizing and secreting ApoE.^54,55^ In addition, a cell culture model of drusen biogenesis developed by Johnson et al. using primary human RPE cells exposed to human serum that mimics several aspects of early AMD showed accumulation of drusen-like deposits that contain ApoE and other drusen-associated proteins.^56^

Genetic variants are strong contributors to AMD accounting for 28%–43% of AMD disease risk depending on the estimated prevalence of AMD in the population.^18,57^ Variants in genes involved in lipid metabolism include *APOE.* ApoE exists as 3 isoforms that differ at 2 amino acid positions: ApoE2 (*Cys112*, *Cys158*), ApoE3 (*Cys112*, *Arg158*), and ApoE4 (*Arg112*, *Arg158*).^58^ The *APOE2* isoform is associated with increased risk for AMD while the *APOE4* isoform is mildly protective against AMD.^59–62^ Variants in other genes involved in lipid metabolism that are associated with AMD risk include hepatic lipase (*LIPC*), cholesteryl ester transfer protein (*CETP*), and lipoprotein lipase (*LPL*), which are involved in HDL cholesterol metabolism, and ATP-binding cassette subfamily A member 1 (*ABCA1*) (Fig. 1).^63–65^ The importance of HDL cholesterol metabolism in the RPE is highlighted by a recent phenotypic study of mice with RPE-specific ablation of *ABCA1* and ATP-binding cassette transporter G1 (*ABCG1*).^66^ The RPE-specific ablation of *ABCA1* but not *ABCG1* was sufficient to cause lipid accumulation in the RPE, degeneration of the RPE and photoreceptors, and visual loss in mice.^66^ Interestingly, the AMD-associated genetic variant of *ABCA1* decreases its expression in RPE cells.^66^ ABCA1 and ABCG1 are critical exporters of cholesterol from cells,^67^ suggesting the molecular explanation of the risk associated with ABCA1 variants and AMD may be due to decreased export of cholesterol from RPE cells.

Environmental and lifestyle stressors modulate the effects of aging and genetic variants on AMD development. Cigarette smoking strongly influences the risk for AMD, whereas obesity, hypertension, sunlight exposure, and alcohol consumption are mildly associated with disease risk.^68–73^ A high-fat (HF) diet is an established risk factor for AMD^74–76^ and often used in AMD mouse model studies.^20,21,77–84^ Consuming diets with a high concentration of fish oils containing omega-3 fatty acids are related with a decreased incidence of AMD^85–88^ compared with increased incidence of AMD in individuals eating diets high in omega-6, monounsaturated, polyunsaturated, and trans unsaturated fatty acids (Fig. 1).^86,87^ It is largely unknown how these environmental stressors interact with advanced aging and genetics to influence AMD pathogenesis.

## Models of AMD

AMD can be modeled using a variety of organisms that range from zebrafish to mice to nonhuman primates. Nonhuman primates are ideal models due to the presence of a macula, drusen in aged animals,^89^ and shared common AMD susceptibility genes such as age-related maculopathy susceptibility 2 (*ARMS2*)/HtrA serine peptidase 1 (*HTRA1*),^90^ but these animals are very costly to maintain and have a slow disease progression. Nonmammalian models like zebrafish are advantageous in that they produce large quantities of offspring and allow for easy assessment of eye phenotypes due to their transparent bodies in juveniles, but they differ from mammals in their retinal vasculature organization and develop photoreceptor degenerations beginning in the larval stage and not in the adult fish.^91,92^

Mice are the most routinely used model organisms for studying AMD due to their short life span, genetic and pharmacologic manipulability, inexpensive housing, and retinal architecture that is similar to humans. The mouse retina is particularly susceptible to the development of age-dependent retinal pathologies, such as decreased visual function,^93,94^ BLamD formation,^95^ RPE multinucleation,^96^ cataract formation,^97^ ectopic synapse development,^98^ and neuroinflammation,^98,99^ validating their usage in interrogating mechanisms associated with age-dependent retinal diseases like AMD.

**FIG. 2.**
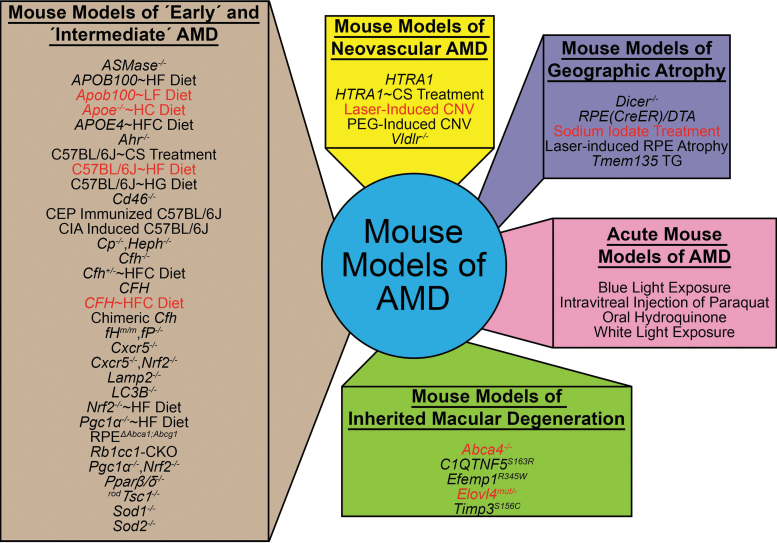
Mouse models of AMD. AMD mouse models can be divided up into 5 categories, including mouse models of “early” and “intermediate” AMD, mouse models of neovascular AMD, mouse models of geographic atrophy, acute mouse models of AMD, and mouse models of inherited macular degeneration. Mouse models highlighted in *red* are the ones used to test the role of lipids in AMD-like pathology development. For more information on these models, we refer readers to Table 1.

The most commonly used mouse models in AMD research largely represent early and intermediate dry AMD, although there are a few models that aim to model wet or late dry AMD (Fig. 2 and Table 1). One important consideration for an AMD mouse model is the incorporation of advanced aging, as aging is the strongest risk factor for human AMD. Many early and intermediate dry AMD mouse models incorporate advanced aging, thus effectively incorporating the effect of chronic processes that drive the development of early/intermediate AMD pathology.^20,21,77,80,81,83,100–124^ In contrast, models of late AMD stages are often based on acute insults to young mice that consequently develop retinal pathology in a short time frame (e.g., 1 to 2 weeks).^125–127^ Therefore, these models may not faithfully reflect the complex age-dependent pathological cues and/or mechanisms that cause AMD and may confound the interpretation of drug efficacy studies.

**Table 1.** Summary of Current Age-Related Macular Degeneration Mouse Models

	Mouse model	Abbreviation	Comments	References
Mouse models of “early” and “intermediate” AMD	Acid sphingomyelinase (ASMase) knockout (KO) mouse	*ASMase^−^* ^/−^	Deficiency of ASMase leads to age-dependent visual loss, photoreceptor degeneration, and increased RPE autofluorescence that correlates with decreased retinal sphingomyelin levels and increased eyecup autophagy.	^131^
	Human apolipoprotein B100 (*APOB100*) mouse fed a high-fat (HF) diet	*APOB100*∼HF	C57BL/6J mice that contain the full-length human *APOB* gene were fed a HF diet and aged to 12 months but no apparent phenotypic differences were observed between *APOB100* mice fed a normal mouse chow and HF diet.	^77^
	*Apob100* mouse fed a low-fat (LF) diet	*Apob100∼*LF	Mice with *Apob* mutation that prevents the formation of the alternatively spliced *Apob48* variant was used to evaluate LXR agonists.	^100,101^
	Apolipoprotein E (*Apoe*) KO mouse fed a high cholesterol (HC) diet	*Apoe^−/−^*∼HC	4–5-week-old *Apoe^−/−^* mice were fed a HC diet for 25 weeks and had worsened retinal pathologies relative to controls.	^78,79^
	Human apolipoprotein E isoform 4 *(APOE4)* mouse fed a high-fat, cholesterol-enriched (HFC) diet	*APOE4*∼HFC	Aged *APOE4*∼HFC develop more severe AMD-like pathology than aged *APOE2* and *APOE3* mice fed a HFC diet.	^80,81^
	Aryl hydrocarbon receptor *(Ahr)* KO mouse	*Ahr^−^* ^/−^	Chronic mouse model based on AHR activity and protein concentration decreases with age in human RPE cells.^102^	^102^
	C57BL/6J mouse exposed to cigarette smoke (CS)	C57BL/6J∼CS	2-month-old C57BL/6J mice were exposed to cigarette smoke for 5 h a day and 5 days a week for 6 months that resulted in ultrastructural changes to BrM and RPE apoptosis.	^132^
	C57BL/6J mouse fed a HF diet	C57BL/6J∼HF	6-week-old C57BL/6 mice fed a HF diet for 30 weeks developed decreased ERGs, increased fundus abnormalities and thickened BrM.	^82^
	C57BL/6J mouse fed a high glycemic (HG) diet	C57BL/6J∼HG	16-month-old C57BL/6J mice were fed a HG diet until 23.5 months of age and developed more AMD-like pathologies that correlated with changes in the microbiome.^103^	^103,104^
	Complement membrane cofactor protein (*Cd46*) KO mouse	*Cd46^−/−^*	Loss of CD46, a membrane regulator of complement activation, leads to age-dependent mice increases in RPE autofluorescence and BrM thickness as well as decreases in choroidal thickness in mice.	^124^
	C57BL/6J mouse immunized with carboxyethylpyrrole (CEP)-adducted BSA	CEP immunized C57BL/6J	Chronic mouse model with increased inflammasome activation^133^ and increased proinflammatory macrophage infiltration.^134^	^105^
	Collagen-induced arthritis (CIA) in C57BL/6J mouse	CIA Induced C57BL/6J	Mouse model of systemic inflammation that had decreased laser-induced CNV lesion size but worsened RPE pathologies after sodium iodate treatment.	^135^
	Ceruloplasmin (*cp*) and hephaestin (*Heph*) double KO mouse	*Cp^−/−^ Heph^−^* ^/−^	Chronic mouse model in which pathologies result from increased oxidative stress from retinal iron overload as supported by the protection against pathologies with the iron chelator deferiprone.^136^	^106,107^
	Complement factor h (*Cfh*) KO mouse	*Cfh^−^* ^/−^	Absence of CFH leads to excess complement activation resulting in no reservoir of plasma complement proteins and subtle retinal damage in aged *Cfh^−/−^* mice.	^108,109,137^
	Heterozygous *Cfh* KO mouse fed a HFC diet	*Cfh^+/−^∼*HFC	Aged *Cfh^+/−^*∼HFC provides the first multifactorial AMD mouse model suited to test the role of complement components on AMD-like pathology development such as C5a.^138^	^21^
	Human complement factor H *(CFH)* Y402 and H402 mouse	*CFH,Cfh^−/−^*	Transgenic mice expressing the full-length human *CFH* gene encoding either the Tyrosine (Y) or Histidine (H) at amino acid 402 were crossed to *Cfh^−/−^* mice to generate mice that produce only human CFH Y402 or H402 protein, respectively, which functions with mouse complement proteins.	^109^
	Human complement factor H *(CFH)* Y402 and H402 mouse aged and fed a HFC diet	*CFH*∼HFC	Phenotypic differences in ocular phenotypes, circulating lipoproteins and ocular lipoproteins were noted between mice expressing equal concentrations of either the CFH Y402 or H402 variant. Only *CFH-H/H* develop AMD-like phenotype	^20^
	Chimeric, transgenic *Cfh* mouse	Chimeric *Cfh*	Mouse *Cfh* was genetically modified to include short consensus repeat domains 6 through 8 of human *CFH* and included either the Y402 or H402 amino acid but no difference in phenotype was observed between mice expressing these variants.	^110^
	Mutant *Cfh* and Complement factor P (*fP*) KO mouse	*fH^m/m^, fP^−/−^*	Mice with a premature stop codon in the end of exon 19 of the mouse *Cfh* gene that results in complement activation^139^ crossed to *fP* KO mice generated increased sub-RPE basal deposits at an early age.	^140^
	C-X-C chemokine receptor type 5 (*Cxcr5*) KO mouse	*Cxcr5^−/−^*	*Cxcr5^−/−^* mice have increased sub-RPE deposits containing amyloid beta and C3a^111^ that may result from disrupted PI3K/AKT signaling and FOXO1 upregulation.^112^	^113^
	Cxcr5 and nuclear factor-like 2 (*Nrf2*) double KO mouse	*Cxcr5^−/−^*,*Nrf2^−/−^*	Combined deficiency of *Cxcr5* and *Nrf2* worsens AMD-like pathologies observed in both *Cxcr5* and *Nrf2* KO mice.	^141^
	Lysosome-associated membrane protein-2 (*Lamp2*) KO mouse	*Lamp2^−/−^*	Absence of LAMP2 in mice leads to the acceleration of sub-RPE basal laminar deposits that contain extracellular matrix proteins, lipoproteins, and cholesterol.	^114^
	Microtubule-associated protein 1 light chain 3 B (*LC3B*) KO mouse	*LC3B^−/−^*	*LC3B* KO mice have increased phagosomes, decreased fatty acid oxidation, RPE lipid accumulation, and subretinal immune cell infiltration.	^142^
	*Nrf2* KO mouse fed a HF diet	*Nrf2^−^*^/−^∼HF	12-month-old *Nrf^−/−^* mice fed a HF diet for 16 weeks developed a more robust AMD-like phenotype that correlated with interleukin 17-producing γΔ T cells.	^83^
	Heterozygous peroxisome proliferator-activated receptor-γ coactivator 1α (*Pgc1α*) KO Mouse Fed a HF Diet	*Pgc1α^+/−^*∼HF	Combined *Pgc1α* heterozygosity and consumption of a HF diet leads to loss of choroidal fenestrations and increased expression of drusen-associated genes.	^84^
	*Pgc1α* and *Nrf2* double KO mouse	*Pgc1α ^−/−^,Nrf2^−/−^*	Combined deficiency of *Pgc1α* and *Nrf2* causes RPE abnormalities and subRPE basal deposits that correlate with damaged mitochondria and increased ER stress.	^143^
	Peroxisome proliferator-activated receptor*-*β/δ (*Pparβ/δ*) KO mouse	*Pparβ/δ^−/−^*	*Pparβ/δ* deficiency leads to increased lipid accumulation and thickened BrM but attenuates laser-induced CNV lesion size.	^123^
	RPE-specific ATP-binding cassette transporter A1 (*ABCA1*) and ATP-binding cassette transporter G1 (*ABCG1*) mouse	*RPE* ^Δ*Abca1;Abcg1*^	Absence of ABCA1 and ABCG1 in the RPE leads to increased lipids within the RPE, causing RPE dysmorphogenesis, neuroinflammation, and photoreceptor degeneration.	^66^
	RPE-specific KO of RB1-inducible coiled-coil 1 (*Rb1cc1*) mouse	*Rb1cc1*-CKO	Loss of RB1CC1 in mouse RPE leads to autophagy defects and pathologies, including RPE degeneration, subretinal immune cell infiltration, subRPE deposition of inflammatory and oxidatively damaged proteins, subretinal drusenoid deposits, and CNV that precedes neural retinal abnormalities.	^144^
	Rod-specific tuberous sclerosis complex 1 (*Tsc1*) KO mouse	*^rod^Tsc1^−/−^*	Mice with rod-specific *Tsc1* ablation that leads to constitutive activation of mTORC1 and pathologies resembling those observed in early, late dry, and wet AMD.	^122^
	Superoxide dismutase 1 (*Sod1*) KO mouse	*Sod1^−^* ^/−^	Chronic mouse model with a susceptibility to oxidative stress damage as shown by increased retinal damage after an intravitreal injection of paraquat.^145^	^115,116^
	Superoxide dismutase 2 (*Sod2*) knockdown mouse	*Sod2^−/−^*	Chronic mouse model where *Sod2* has been knocked down using a viral-delivered ribozyme and Cre-LoxP recombination that has been used to test the efficacy of RPE65-programmed bone marrow-derived cells *in vivo*.^146^	^117,118^
Mouse models of neovascular AMD	Htra serine peptidase 1 (*HTRA1*) overexpressing mouse	*HTRA1*	HTRA1 overexpression was achieved by RPE65 promoter-driven mouse *Htra1*,^129^ CMV-BEST1 hybrid promoter-driven human *HTRA1*^128^ and CAG-driven mouse *Htra1*^119^ expression in wild-type mice and resulted in pathologies after 1 year of age	^119,128,129^
	*Htra1* overexpressing mouse exposed to cigarette smoke (CS)	*HTRA1*∼CS	12-month-old *HTRA1* overexpressing mice were exposed to cigarette smoke for 30 min per day, 5 days per week for 12 weeks, and resulted in increased CNV and sub-RPE basal deposits.	^119^
	Laser-induced CNV		Commonly used acute model that has been interrogated in multiple transgenic mice (i.e., *Cfh^−/−^*,^137^ *Ahr^−/−^*^147^, and *Cx3cr1^−/−^*^148^ and used for testing novel therapies for wet AMD.	^127,149^
	Polyethylene glycol (PEG)-induced CNV		Subretinal injection of PEG, a complement activator, leads to CNV in mice 5 days postinjection and is dependent on complement activation.	^150^
	Very-low-density lipoprotein receptor (*Vldlr*) KO mouse	*Vldlr ^−/−^*	Mouse used to model retinal angiomatous proliferation.	^151^
Mouse models of geographic atrophy	*Dicer* knockdown mouse	*Dicer ^−/−^*	Acute mouse model where *Dicer* has been knocked down by Cre-LoxP recombination and results in increased *Alu* mRNA and frank RPE cell death mediated by inflammasome activation.^152^	^120,153^
	Inducible Cre recombinase driven by the monocarboxylate transporter 3 promoter and Diphtheria toxin A (*DTA*) with LoxP-flanked stop codon double transgenic mouse	RPE(CreER)/DTA	RPE(CreER)/DTA mice have 60%–80% RPE cell death that results in ERG and retinal pathology and serves as a valuable model for stem cell-derived RPE transplantation studies.	^154^
	Sodium iodate treatment		Acute insult causing RPE damage resulting in RPE atrophy and death, retinal degeneration, and immune cell recruitment by 3 days postinjection.	^125,126,155^
	Laser-induced RPE atrophy		Acute mouse model with focal atrophic photoreceptors, abnormal RPE and BrM, visual loss, and neuroinflammation without signs of neovascularization.	^156^
	Transmembrane protein 135 (*Tmem135*) transgenic (TG) mouse	*Tmem135* TG	Overexpression of *Tmem135* leads to fragmented mitochondria in RPE cells as well as progressive RPE degeneration and dysmorphogenesis without affecting visual function until 1 year of age in mice.	^121^
Acute models of AMD	Blue light exposure		Acute insult that results in damage to the neural retina that has been used to differentiate microglia and bone marrow-derived macrophages in the retina.^157^	^158–160^
	Intravitreal injection of paraquat		Acute insult that results in increased oxidative stress damage and subsequent damage to the neural retina that is used for antioxidant therapy studies.	^161^
	Oral hydroquinone		16-month-old C57BL/6J female mice were given oral hydroquinone in their drinking water that resulted in the development of sub-RPE basal deposits.	^162^
	White light exposure		BALB/c albino mice treated with white light for 24 h leads to photoreceptor apoptosis and visual loss^163^ as well as immune cell infiltration that is worsened after CEP immunization.^134^	^163^
Mouse models of inherited macular degeneration	ATP-binding cassette, sub-family A (*ABC1*), member 4 (*Abca4*) KO mouse	*Abca4^−^* ^/−^	Mouse model of recessive Stargardt's disease that is often used in studies on complement activation,^164^ lipofuscin, and novel drug development for AMD.^165^	^166,167^
	C1q and tumor necrosis factor related protein 5 (*C1QTNF5*) serine to arginine at amino acid 163 (S163R) knockin mouse	*C1QTNF5^S163R^*	Mouse model of late-onset retinal degeneration generated by an introduction of the S163R mutation into the mouse *C1QTNF5* gene^168^ as well as viral delivery of human *C1QTNF5^S163R^* to the RPE of C57BL/6J mice.^169^	^168,169^
	EGF-containing fibulin-like extracellular matrix protein 1 (*Efemp1*) arginine to tryptophan at amino acid 345 (R345W) knockin mouse	*Efemp1^R345W^*	Mouse model of Malattia Leventinese/Doyne's Honeycomb Dystrophy revealed a vital role of complement in sub-RPE deposit formation^170^ and replicated using cell culture.^171^	^172,173^
	Elongation of very-long-chain fatty acids protein 4 (*Elovl4*) five base pair deletion knockin mouse	*Elovl4^mut/−^*	Mouse model of dominant Stargardt's disease characterized by defects in very-long-chain polyunsaturated fatty acids in the retina.^174^	^175,176^
	Metalloproteinase inhibitor 3 (*Timp3*) serine to cysteine at amino acid 156 (S156C) knockin mouse	*Timp3^S156C^*	A mouse model of Sorsby's dystrophy used in angiogenesis studies.^177^	^178^

AMD, age-related macular degeneration; BrM, Bruch's membrane; CNV, choroidal neovascularization; ERG, electroretinography; LXR, liver X receptor; mTOR, mammalian target of rapamycin; RPE, retinal pigmented epithelium.

One exception is the wet AMD model using aged *HTRA1*-overexpressing mice where pathologies were observed at 1 year of age.^119,128,129^ An important issue to consider is that AMD-associated variants at chromosome 10q26 loci, where *HTRA1* is located, have been shown to decrease *HTRA1* expression, suggesting *HTRA1*-overexpressing mice may not represent the role of HTRA1 in AMD pathobiology.^130^ This is further supported by a recent article from the Hageman group showing that HtrA1 is specifically reduced as much as 50% in the RPE of humans with the *ARMS2* risk allele.^130^ This appears to be due to disruption of a cis-acting regulatory element within the *ARMS2* locus further supporting that augmentation, not inhibition, of *HTRA1* is a rational therapeutic approach for AMD patients with the 10q26 risk allele.^130^

A popular strategy for generating AMD mouse models has been to subject them to acute insults aimed at reproducing AMD-relevant environmental stressors and assessing retinal damage after a few days and/or weeks. Because oxidative stress has been implicated in AMD, many of these acute insults increase the oxidative stress burden in the eye. These include systemic sodium iodate treatment,^125,126^ intravitreal paraquat,^161^ oral hydroquinone,^162^ and blue light exposure^158–160^ (Fig. 2 and Table 1).

The most commonly used acute AMD mouse model is laser-induced CNV, which is meant to model wet or neovascular AMD^127^ (Table 1). Importantly, it has been a reliable animal surrogate in the development of therapeutics to treat wet AMD, including predicting the clinical efficacy of anti-VEGF therapy for wet AMD.^149^ Laser photocoagulation is used to disrupt BrM in laser-induced CNV, stimulating growth of new choroidal blood vessels toward the retina.^127^ The laser-induced CNV mouse model produces quick results within a few weeks, but it is more of a wound-healing model.^179^ Unlike in human CNV, the laser-induced CNV in rodents spontaneously regresses after a few weeks and there is considerable variation in outcome between mouse strains, genotypes, and age.^180^ Still, the laser-induced CNV mouse model has been widely used in studies evaluating therapies targeting components of lipid metabolism for exudative AMD, such as omega-3 long-chain polyunsaturated fatty acids (LCPUFAs),^181–184^ apolipoprotein A-I (ApoA1) and ApoAI-binding protein (AIBP),^185^ apolipoprotein M (ApoM),^186^ HDL eye drops,^187^ AREDS2 supplementation,^188^ atorvastatin,^189^ pitavastatin,^190^ and cytochrome P450 oxidase 2C (CYP2C) inhibition.^191^

The use of mouse models of inherited macular degenerations may be advantageous to test preclinical AMD therapeutics due to their quicker onset, robust penetrance, and severe pathology development. A subset of mouse models were developed based on mutations in genes that cause inherited macular degenerations in humans, including C1q and tumor necrosis factor-related protein 5 (*C1QTNF5*) in late-onset retinal macular degeneration,^168,169^ EGF-containing fibulin-like extracellular matrix protein 1 (*EFEMP1*) in Malattia Leventinese/Doyne's honeycomb retinal dystrophy,^172,173^ metalloproteinase inhibitor 3 (*TIMP3*) in Sorsby's fundus dystrophy,^178^ and ATP-binding cassette subfamily A member 4 (*ABCA4*) in autosomal recessive Stargardt disease (STGD1) or elongation of very-long-chain fatty acid protein 4 (*ELOVL4*) in autosomal dominant Stargardt macular dystrophy (STGD3)^166,167,175,176^ (Fig. 2 and Table 1). Inherited macular degenerations differ from AMD since they are caused by a genetic mutation and generally present earlier in life.^192–196^ Still, regardless of the different etiologies between these diseases, the mouse models of these macular degenerations recapitulate many of the cardinal features of their respective inherited macular degeneration and some features of AMD.^166,168,172,173,175,176,178,197,198^

## Limitations of Mouse Models in AMD and Lipid Metabolic Research

Although there are many advantages to using preclinical AMD mouse models, it is critical that AMD researchers be well informed of the potential confounders that might influence the interpretation of AMD mouse model studies. For example, there are genetic mutations within some mouse strains that can cause retinal degenerations such as the *rd8* mutation in Crumbs homolog 1 (*Crb1*)^199^ and *rd1* mutation in phosphodiesterase 6B (*Pde6b*).^200,201^ Before beginning any study, researchers should screen their mice to confirm the absence of these mutations in their colonies. Another important consideration of AMD mouse models is the genetic background of the mouse. It is becoming apparent that the phenotypes of murine disease models are heavily dictated by their mouse genetic background as observed with mouse models of Alzheimer's disease.^202^ Lastly, environmental variation can also exert unwanted effects on AMD mouse model studies. Differences in normal mouse chow, microbiota, and stress levels can affect phenotypic outcome measures.^203^ Consideration, careful planning, and appropriate controls are crucial determinants for the success of any study using AMD mouse models. Here are some of the disadvantages of using preclinical AMD mouse models based on differences in the anatomy of their visual system and lipid metabolism.

### Differences between mouse and human retinas

Notable distinctions exist between the mouse and human retina. For the most part, mouse and human eyes undergo similar early developmental programs, but these programs diverge to allow for the complex structure of the human retina.^204^ Importantly, mice do not possess a cone-dominant macular region but rather a rod-rich retina that is similar to the rod-rich parafovea in humans.^205^ This similarity is an advantage of using the mouse retina for modeling early to intermediate AMD since this rod-rich region of the human macula is where macular degeneration is first detected.^206^ The cone-dominant macular region in the human retina allows for high visual acuity, but mice lacking this type of retinal structure have extremely low visual acuity that is equivalent to 20/2,000.^207^ Rods and cones have different energy demands^208^ and transcriptional profiles,^209^ thus, there could be difficulty in translating findings of ophthalmic drug studies in mice to humans.

A potent physiological difference between a mouse and human retina is the interaction between photoreceptors and RPE cells. RPE cells are tasked with delivering nutrients to photoreceptors and ingesting photoreceptor outer segment wastes.^210^ The central part of the mouse retina contains a higher density of photoreceptors than the central part of the human retina, equating to a higher proportion of photoreceptors to RPE cells in the central murine retina.^205^ In addition, there are more smaller and denser RPE cells in the center of the mouse retina than the central human retina.^205^ These differences suggest that murine RPE may have increased phagocytosis of photoreceptor outer segments and may explain transcriptional differences between mouse and human RPE, including increased expression of oxidative stress and outer retinal barrier genes.^211^ It has been shown that mice have higher basal metabolic rates than humans^212^ and could suggest higher metabolic rates in mouse RPE cells than human RPE cells. Furthermore, since RPE cells have coordinated metabolic relationships with both rod and cone photoreceptors,^213^ it is plausible that there are variations in the metabolic ecosystem within the murine retina versus human retina and may confound therapeutic studies, especially those focusing on metabolism.

### Differences between mouse and human lipid metabolism

Mice and humans have similar expression of genes involved in lipid metabolism within the retina,^214,215^ but it is unknown if there are differences between lipid metabolic functions between mouse and human RPE. One important expression difference between these species is the absence of *CETP* in the mouse.^216^ CETP facilitates the transfer of cholesterol between HDL to very-low-density lipoprotein (VLDL) and LDL.^217^ Because of the CETP deficiency in mice, most plasma cholesterol is confined to HDL particles.^218^ This is in stark contrast to humans where most plasma cholesterol is found in LDL particles.^218^ Recapitulating the plasma lipid profiles of humans in mice can be achieved by diet intervention but often these diets do not represent typical diets consumed by humans. Strikingly, regardless of the absence of CETP and diet intervention, both mice and humans have comparable lipoprotein proteomes that may indicate similar functions of lipoproteins between mice and humans.^218^ Lastly, differences in transcription factors involved in lipid metabolism between mice and humans may influence lipid-targeting drug studies in AMD mouse models.^219^ Thus, caution is warranted when extrapolating conclusions from lipid metabolism, notably with regard to cholesterol metabolism, studies in mice to humans.

### Translatability of mouse studies to the clinic

**FIG. 3.**
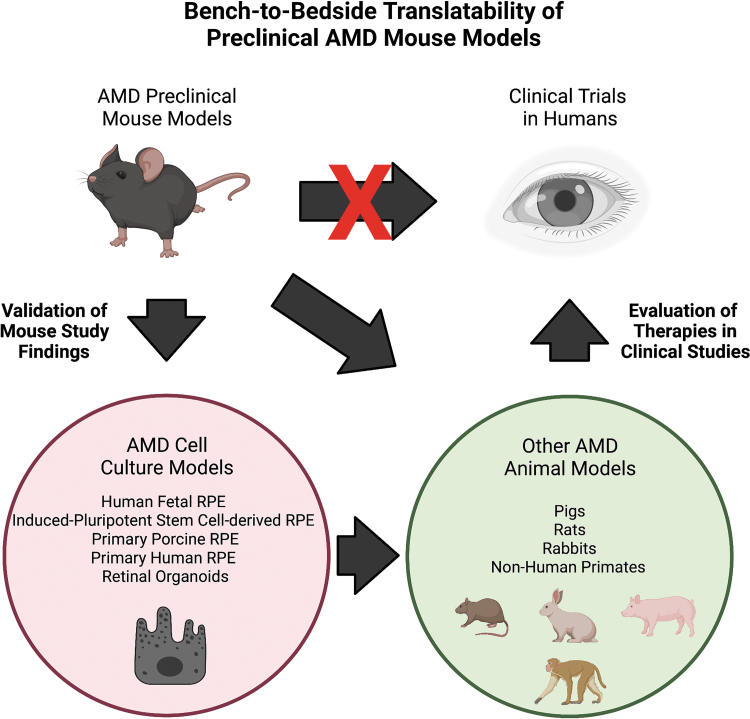
Bench-to-bedside pipeline of AMD therapeutics. Preclinical AMD mouse models can be used to evaluate therapeutics for AMD but these therapeutics need to be tested in cell cultures modeling AMD and/or higher order animal models of AMD. Then, promising therapies can advance to clinical trials to determine if they are viable treatments for AMD. Multiple models should be utilized to translate preclinical AMD mouse model studies to clinically relevant applications.

The anatomical differences between the mouse and human retina have called into question the usefulness of mice in evaluating therapies for AMD since these studies may not translate well to the clinic. However, it is important to remember that AMD mouse models are only 1 step in the bench-to-bedside pipeline for translating basic research to clinical treatments (Fig. 3). We strongly advocate for the validation of preclinical AMD mouse model studies with other models of AMD. In parallel with the mouse models presented in this review, advances have been made in cell-culture systems such as fetal human RPE,^220^ induced-pluripotent stem cell-derived RPE,^221^ primary porcine RPE,^222^ and retinal organoids^223^ that allow for confirmation of mouse study findings in human cells (Fig. 3). Ultimately, any therapeutic showing promising effects should be tested in higher order animal models of AMD, including rats, rabbits, pigs, or nonhuman primates (Fig. 3). No model of AMD has represented the full complex spectrum of AMD but combining multiple models together is essential for the translating results from mouse studies to humans.

## Devising and Testing Lipid Metabolism-Targeting Therapies in Preclinical AMD Mouse Models

**FIG. 4.**
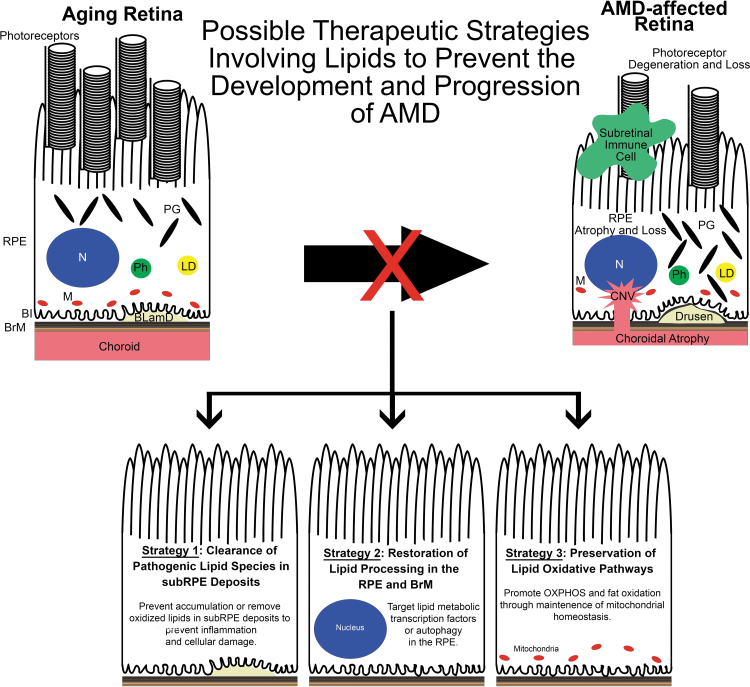
Possible therapeutic strategies involving lipids to prevent AMD development and progression. Based on previous AMD mouse model studies, we propose 3 therapeutic strategies involving lipids. *Left:* Targeting pathogenic lipid species in sub-RPE deposits by preventing their accumulation or removing them may reduce their ability to induce an inflammatory response in the sub-RPE milieu, to cause RPE damage, and may impede the development of AMD. *Middle:* Lipid homeostasis in the RPE is modulated by the actions of various transcription factors such as SREBPs, LXRs, and RXRs as well as autophagy. It is known that aging disrupts the signaling associated with these transcription factors and autophagic processes. Restoring these pathways may serve as valuable therapeutics in preventing AMD. *Right:* Mitochondria are important regulators of lipid oxidation. With age, mitochondria number and function decline in the RPE that may predispose the eye to AMD. By preserving or augmenting mitochondrial function, the RPE may remain healthy and avoid the dysfunction seen in AMD. LXRs, liver x receptors; RXRs, retinoid X receptors; SREBPs, sterol regulatory element-binding proteins.

Numerous AMD mouse models are available to researchers, but it can be a daunting task to best leverage these models to design and determine drug efficacy of therapies targeting lipid metabolism. We propose 3 major therapeutic goals based on previously published AMD mouse model studies: (1) clearance of pathogenic lipid or protein components in sub-RPE deposits, (2) restoration of lipid processing in the RPE and BrM, and (3) preservation of lipid oxidative pathways (Fig. 4). In this study, we will discuss support for these strategies.

### Clearance of pathogenic lipid or protein components in Sub-RPE deposits

Testing therapies directed at preventing the formation and/or accumulation of BLamDs in AMD mouse models is a good use of these models with the caveat that these deposits are not completely analogous to pathogenic drusen in AMD,^21,77,80,102,104,105,109,110,117–119,128,132,224^ such that therapies that work in these preclinical models may not necessarily translate well to human clinical trials. In support of this approach, it is important to note that many drusen constituents are found in BLamDs, including complement components,^21,81,83,105,106,108–110,118,148,224^ apolipoproteins,^81^ amyloid beta,^81^ extracellular membranous debris,^104,109,224^ and long-spaced collagen.^77,102,107,109^ Targeting specific components within BLamDs has led to amelioration of AMD-like pathologies in AMD mouse models. For example, aged transgenic *APOE* mice with targeted replacement of mouse *ApoE* with human *APOE4* develop an AMD-like ocular phenotype, including decreased visual function, CNV, RPE damage, and BLamD accumulation after an 8-week high-fat, cholesterol-enriched (HFC) diet.^80^ *APOE4* is associated with decreased risk for AMD in humans but the pathogenicity caused by the *E4* allele in aged mice fed a HFC diet is unknown.^59–62^

The BLamDs of *APOE4*∼HFC mice contain complement activated products and amyloid-beta.^81^ Targeting amyloid beta with a systemic anti-amyloid-beta immunotherapy prevented amyloid-beta accumulation, sub-RPE complement activation, and AMD-like pathologies, although there was no change in BLamD load.^81^ Preventing the accumulation of toxic inflammatory components within drusen-like amyloid beta may be a viable therapeutic approach for AMD. However, a humanized monoclonal antibody against amyloid beta was tested in a clinical trial for the treatment of geographic atrophy but it failed to slow the geography atrophy enlargement in patients,^225^ indicating not all therapies assessed in mice are translatable to human diseases. Still, it should be noted that the anti-amyloid antibody, RN6G, which showed efficacy in the mouse and targets the c-terminus of both Ab40 and Ab42 was not the one tested in clinical trials.^81^

Similar to amyloid beta, oxidized lipids can be pathogenic to RPE cells.^26^ A therapeutic strategy for targeting inflammatory lipids in AMD has emerged from studies of AMD mouse models with varying complement factor H (CFH) activity.^20,21,138^ *CFH* is a major AMD susceptibility gene^226–229^ that functions as the main soluble regulator of the alternative complement pathway by serving as a cofactor for factor I-mediated proteolytic inactivation of C3b^230^ and accelerating the decay of the C3 convertase that is responsible for the intial activation and propagation of the complement cascade.^231^ The importance of CFH in regulating the formation of the C3 convertase is exemplified by the absence of intact plasma C3 in *Cfh* knockout (*Cfh^−/−^*) mice due to uncontrolled C3 cleavage.^21,232^

In addition to its canonical functions, we have found that CFH can bind and decrease adherence of ApoE- and ApoB-containing lipoproteins to BrM.^21^ To examine the significance of impaired CFH binding to lipoproteins *in vivo*, C57BL/6J, *Cfh* heterozygous (*Cfh^+/−^*), and *Cfh^−/−^* mice were aged to ninety weeks and then fed a HFC diet for 8 weeks to exacerbate the subtle AMD-like phenotype seen in aged *Cfh^−/−^* mice.^21,108,109^ Consumption of a HFC diet leads to increased circulating lipoproteins in mice.^233^ Both aged *Cfh^+/−^* and *Cfh^−/−^* mice accumulate sub-RPE basal deposits in response to the HFC diet.^21^ Notably, only the aged *Cfh*^+/−^ and not the *Cfh^−/−^* null mice fed a HFC diet, developed sustained vision loss and RPE damage.^21^ Aged *Cfh^−/−^* mice may be protected against HFC-induced ocular damage from pathogenic sub-RPE basal deposits because they lack a reservoir of complement components and they display increased expression of membrane-bound complement regulators in the posterior eye, whereas *Cfh^+/−^* mice possess an intact complement system, but express only half the levels of Cfh as wild-type mice.^21,234,235^

CFH also acts to regulate RPE-derived lipoprotein accumulations in BrM. Evidence for a unique RPE-derived lipoprotein comes from biochemical assessments of human donor eyes.^23,236^ RPE cells possess the machinery required to generate lipoproteins^237^ and have been validated by detecting secreted lipoproteins in the media of RPE cell cultures.^236–238^ Our recent study of transgenic mice expressing equal amounts of the full-length normal human CFH Y402 versus the AMD-risk-associated CFH H402 variant on a *Cfh* null background (*CFH-Y/0* and *CFH-H/H*, respectively) revealed a correlation between RPE-derived lipoproteins and pathologies.^20^ The *CFH Y402H* polymorphism is one of the most replicated genetic variants associated with AMD risk,^226–229^ so these mice were developed to test the *in vivo* effect of the Y402H CFH risk variant on AMD pathobiology.^20,109^ *CFH-Y/0* and *CFH-H/H* mice were aged to 90 weeks on a normal mouse chow diet (ND) and then switched to a HFC diet for 8 weeks to test for an AMD phenotype as aging and the consumption of a HFC diet are sufficient to elicit AMD-like pathology development in *Cfh* heterozygous mice and *APOE4*-targeted replacement mice.^21,80^ Only the old *CFH-H/H* mice fed HFC (*CFH-H/H∼*HFC) developed AMD-like pathologies.^20^

Quantitation of plasma lipoproteins in these mice revealed decreases in plasma LDLs and its markers, apolipoprotein B100 (ApoB100), and ApoE in aged *CFH-H/H*∼HFC mice compared with aged *CFH-Y/0* mice fed HFC (*CFH-Y/0*∼HFC), but no change in any other lipoprotein class.^20^ Strikingly, however, biochemical analyses revealed that changes in eyecup apolipoproteins correlated with the AMD-like phenotype seen in the *CFH-H/H*∼HFC, where apolipoproteins B48 (ApoB48) and A1 (ApoA-1) are elevated in the RPE/choroid of the aged *CFH-H/H*∼HFC mice compared with age-matched control *CFH-Y/0* after an 8-week HFC diet.^20^ Thus, we are the first to establish a functional consequence of the Y402H polymorphism *in vivo*, promoting AMD-like pathology and affecting lipoprotein levels in aged mice.^20^

**FIG. 5.**
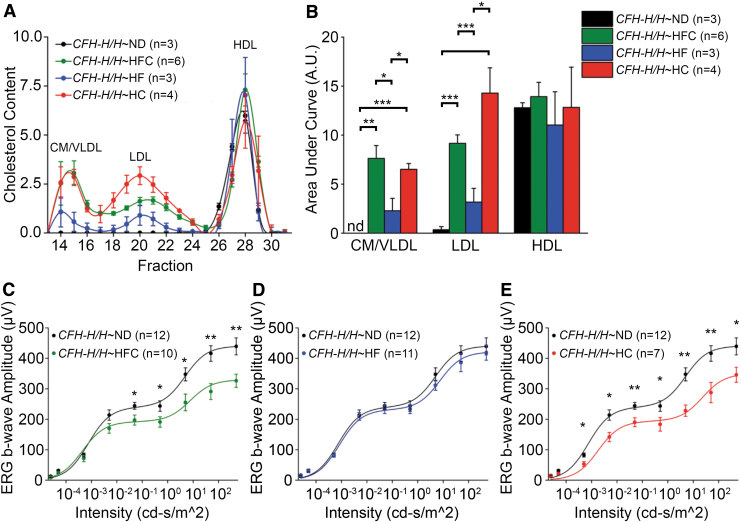
Dietary intervention effects on plasma lipoprotein levels and visual function of aged *CFH-H/H* mice. Male *CFH-H/H* mice over 90 weeks of age, housed conventionally and maintained on ND (Isopurina 5001; Prolab) were either continued on ND or switched to a HFC (Envigo #88051), HF (Envigo #98232) or HC (Envigo #91342) diet for 8 weeks. All mice were negative for the *rd8* mutation. Protocols for FPLC fractionation, cholesterol quantification, ERG, and statistical analysis are described in Landowski et al.^20^ **(A)** FPLC fractions of male aged *CFH-H/H* mice after an 8-week ND, HFC, HF, and HC diet. **(B)** Averages of the area under the FPLC curve for aged male *CFH-H/H* mice after an 8-week ND, HFC, HF, and HC diet. Consumption of dietary cholesterol increases CM/VLDL and LDL cholesterol fractions in aged male *CFH-H/H* mice relative to the ND- and HF-fed groups. **(C–E)** Visual function in aged male *CFH-H/H* mice after an 8-week ND, HFC, HF, and HC diet. Analysis of scotopic ERG b-wave responses reveals statistically lower b-wave amplitudes in aged *CFH-H/H* mice fed a HFC and HC diet compared with ND-fed controls. Data are presented as fitted lines of the average. Mean ± SEM. *P < 0.05, **P < 0.01, and ***P < 0.001. CFH, complement factor H; CM, chylomicron; ERG, electroretinography; FPLC, fast protein liquid chromatography; HC, high cholesterol with no added cocoa butter fat; HDL, high-density lipoprotein; HF, high fat with no added cholesterol; HFC, high-fat, cholesterol-enriched; LDL, low-density lipoprotein; ND, normal mouse chow diet; nd, not detected; VLDL, very-low-density lipoprotein.

The identity of molecular pathways involved in RPE lipoprotein synthesis and secretion are largely unknown, but insight may be gleaned from a dietary intervention study using aged *CFH-H/H* mice performed in our laboratory. Diets with similar macronutrient composition as the HFC diet previously used, which either had the dietary cholesterol removed (HF) or the fat removed [high cholesterol (HC)], were used to test the role of dietary cholesterol and fat on circulating lipoprotein and visual function in aged *CFH-H/H* mice. Aged *CFH-H/H* mice consuming a HFC or HC diet have increased chylomicron (CM)-, VLDL-, and LDL-containing cholesterol compared with aged *CFH-H/H* mice after a ND or HF diet (Fig. 5A). The area under each lipoprotein curve was calculated and the confirmed levels of these lipoproteins were statistically different between these groups (Fig. 5B). Visual function of aged *CFH-H/H* mice was assessed by scotopic electroretinography (ERG) after the consumption of an 8-week HF or HC diet.^81^ Using another cohort of aged *CFH-H/H* mice, these mice developed a similar decrease of scotopic ERG b-wave responses after the consumption of an 8-week HFC diet (Fig. 5C), as previously described.^20^ No change was observed in aged *CFH-H/H*∼HF mice relative to aged *CFH-H/H*∼ND mice (Fig. 5D). Notably, aged *CFH-H/H*∼HC mice developed significantly attenuated ERG b-wave responses compared with aged *CFH-H/H*∼ND mice (Fig. 5E).

The correlation of decreased visual function with consumption of dietary cholesterol in aged *CFH-H/H* mice fed a HC diet but not when fed a HF diet, supports the notion that therapeutic strategies targeting cholesterol intake through the small intestine (such as ezetimibe) may prevent visual loss in aged *CFH-H/H*∼HFC mice. Ezetimibe interacts with the Neimann Pick C1-like intracellular cholesterol transporter 1 (NPC1) to prevent cholesterol uptake by intestinal enterocytes and thereby lowering the level of plasma LDLs containing cholesterol.^239^ Ongoing studies are examining whether ezetimibe could mimic the effects of dietary modulation in aged *CFH-H/H* mice and if HFC diet-induced increases in eyecup ApoB48 and ApoA-1 in aged *CFH-H/H* mice is influenced by dietary cholesterol.

ApoA-1 is a major protein constituent of HDL, a lipoprotein associated with AMD risk.^48–51^ In the posterior eye, the major site of interaction between CFH and HDL occurs at heparan sulfation within BrM.^22,240^ We postulate that augmenting CFH concentrations or soluble heparan sulfate in the posterior eye may prevent toxic accumulations of lipoproteins such as HDL in BrM and protect against RPE damage and death. In support of this, treatment of BrM explants from human donor eyes with short heparan sulfate oligosaccharides or an ApoA-1 mimetic was sufficient to remove lipoproteins from BrM.^23^ In addition, aged nonhuman primates that got an intravitreal injection of an ApoA-1 mimetic had less neutral lipid, esterified cholesterol, and activated complement components in BrM than placebo-treated controls.^241^ Pharmaceutical interventions aimed at lipoprotein binding in BrM may be effective in the treatment of AMD and should be considered in future studies involving AMD mouse models.

### Restoration of lipid processing in the RPE and BrM

Restoration of pathways that the RPE cells use to process lipids could be a means to therapeutically target lipid metabolism in AMD. For example, multiple transcription factors, including sterol regulatory element-binding proteins (SREBPs),^242^ liver X receptors (LXRs),^243^ retinoid X receptors (RXRs),^244^ and peroxisome proliferator-activated receptors (PPARs)^245^ participate in intracellular lipid homeostasis. The SREBP, LXR, RXR, and PPAR pathways are present in RPE cells^100,123,215^ and their importance in lipid homeostasis of the RPE is supported by reports of visual loss and lipid deposition in mice lacking LXR and PPAR β/δ signaling.^100,123^

In a study aimed at determining if activating LXRs can prevent AMD-like pathology development, 3-month-old mice with a mutation in apolipoprotein B100 (*apob100*) that prevents the formation of its alternative splice variant apolipoprotein B48 (*apob48*) were fed a low-fat diet for 5 months and given GW3965, an LXR agonist.^100^ Limiting expression to only ApoB100 in mice increased LDL triglyceride and cholesterol levels, resembling lipoprotein profiles in humans.^246^ As a consequence of this metabolic change, age-related progression of lipid deposition occurs in the BrM of *apob100* mice but no other pathologies were noted in these animals.^101^ GW3965 treatment improved hypopigmented regions in fundus images, dampened neuroinflammation, and decreased lipid deposition in the *apob100* mice after the 5-month low-fat diet.^100^ Differences in the ocular phenotypes of the *apob100* mice may be explained by the standard mouse chow and low-fat diet consumed by mice in these 2 studies.^100,101^ More investigations of LXR agonists and other agents modulating transcription factors in other AMD mouse models are needed to confirm the promising results after treating *apob100* mice with GW3965.

Another pathway critical for lipid metabolism within the RPE is autophagy, a conserved catabolic pathway induced under cellular stresses.^247^ RPE cells depend on autophagy for the daily breakdown of photoreceptor outer segments.^144^ Autophagy is increased in RPE cells of aged non-AMD eyes, but decreased in RPE cells of human donor eyes diagnosed with AMD.^248^ Mice with decreased autophagy such as microtubule-associated protein 1 light chain 3 B (*LC3B*) knockout, lysosomal-associated membrane protein *2* (*Lamp2*) knockout,^114^ and RPE-specific RB1-inducible coiled-coil 1 (*Rb1cc1*) knockout mice have lipid accumulations within the RPE.^142,144^ As a consequence, these mouse lines develop visual loss, migration of immune cells into the subretinal space, and subRPE deposits.^114,142,144^ Studies utilizing RPE cell cultures identified a synthetic lignan secoisolariciresinol diglucoside, LGM2605,^249^ and flubendazole^250^ as inducers of autophagy that were able to reduce intracellular RPE lipid levels. Testing these therapies in mice with increased RPE lipid accumulation like RPE-specific *ABCA1* and *ABCG1* knockout mice^66^ could be an effective means testing if targeting autophagy may be a therapy for AMD.

### Preservation of lipid oxidative pathways

Another possible intervention is to sustain the lipid oxidative function of RPE cells through preservation of mitochondrial health. Mitochondria are critical organelles required for the breakdown of fatty acids for ATP production through beta-oxidation^251^ and regulation of reactive oxygen species that, when in excess, leads to lipid peroxidation.^252^ With age, mitochondrial function declines and impacts retinal homeostasis.^33^ Multiple AMD mouse models are based on mitochondrial dysfunction, including the *Tmem135* transgenic (*Tmem135* TG)^121^ and superoxide dismutase 2 knockout (*Sod2^−/−^*).^117,118,253^ It is possible that other mouse models may have mitochondrial impairment such as the DICER1-deficient mice.^120,152,153^ Not only is there a consequence of an accumulation of Alu elements in DICER1-deficient mice, they also have decreased mitochondrial genome-encoded small RNAs and consequent reduced mitochondrial gene expression.^254^ RPE degeneration is a common feature in these AMD mouse models.^117,118,120,121,153,253,254^ The RPE degeneration in these models phenocopies mice with RPE-specific ablation of mitochondrial transcription factor A (*Tfam*)^255^ and global knockout of peroxisome proliferator-activated receptor gamma coactivator 1-alpha (*Pgc1α*),^256^ proteins required for mitochondrial biogenesis.

The RPE-specific *Tfam* knockout mice have increased activation of the mammalian target of rapamycin (mTOR) pathway,^255^ a pathway crucial for nutrient sensing and homeostasis.^257^ Abnormal mTOR activation can cause RPE degeneration^258^ but inhibiting mTOR in mice with RPE-specific ablation of TFAM or in mice treated with sodium iodate, alleviates RPE pathologies.^255^ Furthermore, activation of mTOR signaling through ablation of tuberous sclerosis complex 1 (*Tsc1*) in rod photoreceptors leads to RPE abnormalities.^122^ Conversely, activating mTOR in mouse models of retinitis pigmentosa is sufficient to reduce cone photoreceptor cell death,^259–262^ whereas inhibiting mTOR through rapamycin can decrease retinal vascularization and lead to hypoxia.^263^

One important aspect of the mTOR pathway is its ability to coordinate protein synthesis.^264^ One study mimicked inhibition of the mTOR pathway by administering anisomycin, which curbs eukaryotic protein synthesis, to *rd16* and wild-type C57BL/6J mice.^265^ The *rd16* mice had accelerated retinal degeneration, whereas wild-type mice developed retinal pathologies after anisomycin treatment.^265^ Restoring mitochondrial function, rather than targeting the mTOR pathway, may be a safer avenue for an AMD therapy, as it is less likely to damage other nearby ocular tissues.

## Lipid Metabolism-Targeting Therapies Tested in Preclinical AMD Mouse Models

A fraction of the preclinical AMD mouse models (Fig. 2 and Table 1) has been utilized to evaluate lipid metabolism-targeting therapies. These therapies include desipramine, TO901316, docosahexaenoic acid (DHA), apolipoprotein mimetics, and statins (Table 2). In this study, we present published studies on these therapies as they offer perspectives on potential treatments for AMD.

**Table 2.** Lipid Therapies Tested in Age-Related Macular Degeneration Mouse Models

AMD mouse model	Retinal pathologies observed in model	Pharmacological treatment	Mode of action	Observed effects due to pharmacological treatment	References
5-month-old *Abca4^−^*^/−^	• Delayed dark adaption • Elevated PE in outer segments • Increased A2E in RPE • RPE vacuolization • Increased retinal all-trans-RAL after light exposure • Increased A2E and lipofuscin granules in RPE • Cholesterol and ceramide accumulation in RPE • Increased early endosome number and size • Augmented complement activation	3 Intraperitoneal injections of desipramine for 4 weeks	Inhibitor of ASMase that prevents ceramide production	• Decreased early endosome volume and number in RPE • Prevented C3a activation and signaling in RPE	^274^
		3 Intraperitoneal injections of TO901316 for 4 weeks	LXR agonist	• Decreased early endosome volume and number in RPE	^274^
Various aged *Elovl4^mut/−^*	• Presence of fundus abnormalities • Photoreceptor degeneration • Decreased scotopic ERG a-wave and b-wave • Increased lipofuscin and A2E in RPE • Loss of synapses • Neuroinflammation • Extensive inner retinal remodeling	Dietary DHA supplementation for various lengths of time	C22:6n-3 fatty acid that has anti-inflammatory, anti-angiogenic and antiapoptotic effects	• Preservation of ERG c-wave in 6-month-treated mutant mice • Preservation of cone function in 12-month-treated mutant mice • Decreased A2E levels in 18-month-treated mutant mice	^280a^
10-month-old *Apoe^−/−^*	• Increased implicit time for scotopic b-wave • Decreased ERG oscillatory potential amplitudes • Decreased ONL nuclei number • Thicker BrM • Increased retinal cholesterol	Single intravitreal injection of 4F	APOA1 mimetic with anti-inflammatory and antiatherogenic properties	• Decreased BrM thickness • Decreased esterfied cholesterol deposition in BrM	^290^
Intravenous injection of sodium iodate in 6-week-old C57BL/6J	• RPE degeneration • Photoreceptor degeneration • Neuroinflammation	Dietary HM-10/10 treatment for 3 weeks	APOE/APOJ mimetic possessing antioxidant and anti-inflammatory properties	• Decreased retinal thinning • Prevented photoreceptor degeneration • Reduced cleaved caspase-3 in retina	^291^
6-week-old C57Bl/6J∼30-week HF diet	• Presence of fundus abnormalities • Decrease of scotopic ERG a-wave and b-wave • Accumulation of lipids • RPE vacuolization • Deposition of heterogenous debris below the RPE • Thickened BrM thickness	Dietary simvastatin treatment for 30 weeks	Inhibitor of 3-hydroxy-3-methylglutaryl coenzyme A (HMG CoA) reductase that lowers endogenous cholesterol synthesis	• Decreased prevalance of fundus abnormalities • No accumulation of lipids • Decreased RPE vacuolization • No deposition of heterogenous Debris below the RPE • Decreased BrM thickness	^82^

^a^ No beneficial effect was seen in a different study using a different *Elovl^mut/+^* mouse after a dietary DHA supplementation.^279^

ApoA1, apolipoprotein A-I; APOJ, apolipoprotein J; ASMase, acid sphingomyelinase; DHA, docosahexaenoic acid; PE, phosphatidylethanolamine.

### Despiramine and TO901316

ABCA4 is a member of the ABC transporter family found on rod and cone photoreceptor outer segments.^266–268^ It is responsible for the clearance of all-*trans*-retinal from the disc membranes after phototransduction through the transport of *N*-retinylidene-phosphatidylethanolamine (PE), a product of the reaction of all-*trans*-retinal with PE.^269^ If *N*-retinylidene-PE is not removed from the disc membranes, *N*-retinylidene-PE can react with all-*trans*-retinal to form toxic accumulations of *N*-retinylidene-*N*-retinylethanolamine (A2E) and other bisretinoids in the RPE.^269^ Ablation of the murine *Abca4* gene recapitulates STGD1-like phenotypes in mice, including age-dependent vision loss, delayed dark adaptation, increased retinal PE, deposition of A2E in the RPE, and additional toxic phototransduction byproducts.^166,270,271^

The spatial distribution of lipids in *Abca4^−/−^* RPE detected through a combination of matrix-assisted laser desorption ionization and Fourier transform ion cyclotron resonance imaging mass spectrometry reveals increased bis(monoacylglycerol)phosphate (BMP) lipid species.^272^ BMP lipid species are commonly observed in endosomal/lysosomal storage diseases and regulate cholesterol levels in endosomes.^273^ Kaur et al. reported enlarged early endosomes in the RPE of *Abca4^−/−^* mice that allow more extracellular complement component 3 (C3) intake and activation of the C3-proteolytic cleavage product, C3a.^274^ Inhibiting acid sphingomyelinase with desipramine can decrease the size of early endosomes and prevent C3a activation in *Abca4^−/−^* RPE.^274^ Promoting cholesterol efflux with a LXR agonist, TO901316, can mimic the effects of desipramine on early endosome size in the *Abca4^−/−^* RPE.^274^ Decreasing the size of early endosomes in the RPE may be a viable therapeutic strategy for AMD, although more phenotypic work is needed to determine the functional effects of desipramine and TO901316 on the RPE in *Abca4^−/−^* mice and other models with RPE pathologies.

### DHA treatment

Mutations in *ELOVL4* are known to cause mislocalization and aggregation of ELOVL4 from the endoplasmic reticulum to other organelles in photoreceptors leading to STGD3.^275,276^ ELOVL4 is a fatty acid elongase that preferentially uses eicosapentaenoic acid (EPA) as a substrate to generate very-long-chain polyunsaturated fatty acids (VLC-PUFAs).^277^ To model STGD3, researchers identified a 5 base pair deletion in *ELOVL4* of a STGD3 patient^195^ and generated transgenic mice expressing ELOVL4 with either the same 5 base pair deletion in human *ELOVL4*^175^ or mouse *Elovl4*.^176^ Regarding the efficacy of omega-3 VLC-PUFAs as a treatment for STGD3, a recent long-term clinical study testing diets supplemented with omega-3 VLC-PUFAs, such as EPA and DHA, in STGD3 patients found no changes in the disease progression.^278^ Dietary DHA supplementation was also tested in mice with a mutant *ELOVL4* allele but these studies yielded conflicting results.^279,280^ This could be due to the differential effect of the mutant *ELOVL4* and *Elovl4* allele on VLC-PUFA synthesis in the mouse retina. Only the mice with a mutant *Elovl4* allele displayed a 50% reduction in retinal VLC-PUFAs.^174^ Further studies have confirmed the essential role of ELOVL4 in retinal VLC-PUFA synthesis by ablating *Elovl4* in cone and rod photoreceptors.^281^ Therefore, it would be important to test if DHA supplementation could be a viable therapeutic strategy in other mouse models of disrupted ELOVL4 function. This is especially important since DHA was added to the AREDS2 formulation and could explain why there was no reduction in AMD risk with the AREDS2 formulation.^282^

### Apolipoprotein mimetics

Repurposing lipid-lowering pharmacologic agents that have been effective in other diseases may be beneficial to treat AMD. For example, a major risk factor for cardiovascular disease is increased LDL and decreased HDL.^283^ The opposite has been shown for AMD where increased HDL has emerged as a major risk factor.^48–51^ One possible strategy to treat cardiovascular disease is to promote HDL function through ApoA1 mimetics. HDL particles that harbor ApoA1 are critical for reverse cholesterol transport, which removes lipids from cells and provides anti-inflammatory protection.^284^ ApoA1 mimetics vary in peptide length and promote reverse cholesterol transport and are anti-inflammatory.^285^ However, the effectiveness of ApoA1 mimetics as a cardiovascular disease treatment has been variable with some reports indicating no beneficial changes in patients.^286–288^ and others describing increased HDL function in patients.^289^

To determine if ApoA1 mimetics could be effective against AMD, 10-month-old *Apoe^−/−^* mice were treated with a single intravitreal injection of the ApoA1 mimetic, 4F, and assessed 30 days postinjection for changes in BrM.^290^ 4F mimetic treatment sufficiently lowered esterified cholesterol and prevented ultrastructural changes in *Apoe^−/−^* mice.^290^ In addition, based on our findings of increased ApoA1 in *CFH-H/H*∼HFC mice, we tested the ApoA1 mimetic 5A for 8 weeks in *CFH-H/H*∼HFC and were able to block HFC diet-induced changes in their plasma HDL proteome.^23^ We are currently testing whether the ApoA1 5A mimetic treatment correlate with changes in visual function that occur in both *Apoe^−/−^* and *CFH-H/H* after a HF, cholesterol-enriched diet in these genotypes.^20,78^

Other apolipoprotein mimetics have been tested in preclinical AMD mouse models such as the hybrid ApoE and apolipoprotein J (ApoJ) mimetic, HM-10/10 in the intravenous sodium iodate treatment model of geographic atrophy.^291^ Treatment of C57BL/6J mice with a diet chow containing HM-10/10 after induction of sodium iodate-induced RPE injury was sufficient to partially protect the retina and prevent caspase-3 cleavage in RPE cells.^291^ Since recruitment of immune cells is a major consequence of sodium iodate injury in the retina,^292^ it would be interesting to investigate whether HM-10/10 can reduce sodium iodate-induced immune cell recruitment to the retina as this frequently occurs in AMD mouse models and AMD-afflicted eyes.^293^

### Statins

Pharmaceuticals that could work for AMD and revolutionized the treatment of cardiovascular disease are statins, a class of lipid-lowering agents inhibiting the activity of 3-hydroxy-3-methyl-glutaryl-CoA (HMG-CoA) reductase to increase the uptake of LDLs and decrease plasma cholesterol.^294^ A recent pilot study, which was randomized and placebo controlled, showed that taking simvastatin (40 mg/day) slowed progression of nonadvanced AMD especially for those with the *CFH Y402H* genotype.^295^ Additionally, an open-label prospective pilot multicenter clinical trial found that a high dose of statins given to 26 patients resolved drusenoid pigment epithelial detachments and improved visual acuity without any progression to RPE atrophy or CNV formation.^296^ However, the potency of statins as a treatment for AMD is controversial as evident by varying conclusions of case/control cross-sectional studies where statin usage had a beneficial,^297–299^ worsened,^300,301^ or no effect in AMD.^302–314^

To determine efficacy in an animal model, female C57BL/6 mice were orally treated with simvastatin, atorvastatin, rosuvastatin, and pravastatin at similar concentrations where simvastatin had the highest accumulation in the retina.^315^ Oral gavage of simvastatin led to a 24% reduction in retinal cholesterol content,^315^ suggesting simvastatin can inhibit cholesterol synthesis in the retina. C57BL/6 mice fed a HF diet for 30 weeks were treated concurrently with simvastatin to evaluate the effect of simvastatin on retinal pathology development.^82^ Simvastatin treatment decreased fundus abnormalities and BrM thickness, but did not significantly ameliorate visual function induced by the 30-week HF diet regime.^82^ The absence of a visual function change could be due to an upregulation of cluster of differentiation 36 (CD36), an oxidized LDL receptor critical for ingestion of photoreceptor outer segments,^316^ in the mouse retina.^315^ Intake of oxidized LDL in the posterior eye can lead to increased expression of genes regulating oxidative stress, inflammation, and angiogenesis,^317–320^ as well as an increase in the number of apoptotic RPE cells.^317,321,322^ Further analysis of the C57BL/6∼HF mouse retina may answer whether statins could work as a treatment for AMD.

## Conclusion

AMD is a debilitating blindness with limited therapeutic options but there is hope of new treatments for this age-dependent retinal disease in the future. Over the years, implications of lipid metabolic dysregulation as a key pathobiological mechanism in AMD have emerged. We presented an overview of preclinical AMD mouse models that allow for the establishment of causative relationships with lipids and AMD-like pathology development. The use of these AMD mouse models may lead to worthy lipid-targeting candidate therapies for clinical trials to prevent the blindness caused by AMD.
